# Reactions to infant death by wild vervet monkeys (*Chlorocebus pygerythrus*) in KwaZulu-Natal, South Africa: prolonged carrying, non-mother carrying, and partial maternal cannibalism

**DOI:** 10.1007/s10329-020-00851-0

**Published:** 2020-08-06

**Authors:** Jennifer Botting, Erica van de Waal

**Affiliations:** 1 Inkawu Vervet Project, Vryheid, South Africa; 2 University of Lausanne, Lausanne, Switzerland

**Keywords:** Thanatology, Vervet monkey, Death, Deceased-infant carrying

## Abstract

Observations of dead infant carrying have been reported for many primate species, and researchers have proposed several hypotheses to explain this behaviour. However, despite being a relatively well-studied species, reports of dead infant carrying in wild vervet monkeys *(Chlorocebus pygerythrus)* remain scarce. Here we report 14 observations of dead infant carrying by female vervet monkeys in a population at Mawana Game Reserve, KwaZulu-Natal, South Africa. Most of the females carried the dead infant for a day or less, but one female carried her infant for at least 14 days. In one case the maternal sister of a dead infant carried it after the death of their mother. We also report a case of mother-infant cannibalism: a female consumed part of her deceased infant’s tail. Other post-mortem care-taking behaviours such as grooming, smelling and licking were also recorded. Of 97 recorded infant deaths in this study population since 2010, 14.4% are known to have elicited dead infant carrying, a proportion similar to that reported for other monkey species. We discuss our observations in relation to various hypotheses about this behaviour, including the post-parturition hormones hypothesis, learning to mother hypothesis, and unawareness of death hypothesis.

**Electronic supplementary material:**

The online version of this article (10.1007/s10329-020-00851-0) contains supplementary material, which is available to authorized users.

## Introduction

In recent years, thanatology, the study of death and dying, has received considerable research interest from primatologists (Anderson et al. 2010; Anderson 2017; Watson and Matsuzawa 2018; Gonçalves and Carvalho 2019). By observing how non-human primates (hereafter, primates) react to the death of conspecifics, we can try to understand what they comprehend about the nature of death and dying, as well as examine potential correlates between their reactions to death and our own (Anderson 2016).

One of the most striking primate reactions to death is the response of mothers to the death of their infant. Many authors report primate mothers continuing to direct caretaking behaviours towards their dead infants. This usually involves carrying the infant, and sometimes other caretaking behaviours, such as grooming, vocalizing, swatting away flies, and trying to prevent other group members from taking the corpse (Biro et al. 2010; Fashing et al. 2011).

In a recent review, Gonçalves and Carvalho (2019) collated reports of dead infant carrying in 34 primate species (or subspecies). These reports are unevenly distributed among primate species, with many of the examples from the study of apes (Biro et al. 2010), macaques (Das et al. 2018) or langurs (Rajpurohit 1997). Despite vervet monkeys (*Chlorocebus pygerythrus*) being one of the more intensively studied monkey species (Cheney and Seyfarth 1990; Isbell et al. 2009; McFarland et al. 2014; Teichroeb et al. 2015), reports of dead infant carrying in vervet monkeys are surprisingly lacking. To our knowledge, only one published report on this exists, by Struhsaker (1971), who simply states: “Once a dead infant was carried and handled by its mother and other females in the group for at least 3 days” (p. 239). The report includes a photograph of the dead infant being handled, but no further information.

To add to this sparse literature on dead infant carrying in vervet monkeys, we report 13 observations of mothers carrying recently deceased infants, and one observation of a sub-adult female carrying her deceased infant sister, recorded in Mawana Game Reserve between 2010 and 2019. We describe the various care-taking behaviours shown and compare these to dead infant carrying behaviour seen in other primate species.

## Methods

Observations were made on seven groups of wild vervet monkeys at the Inkawu Vervet Project, Mawana Game Reserve, KwaZulu-Natal, South Africa (28°00.327S, 031°12.348E) between 2010 and 2019. Habituation of the groups began in 2010 (groups AK, BD, NH, LT), 2012 (KB), and 2014 (CR). One group (IN) was a split group consisting of three females formerly belonging to a larger group (BD). The habitats of the groups vary, but all of the groups spend some time in riverine forest, as well as in open savannah and areas covered by large numbers of acacia trees (*Acacia tortillis* and *Acacia nilotica)*. Vervet monkeys at Inkawu Vervet Project generally give birth between September and December, at the start of the rainy season. The temperature ranges from 7 to 24 °C in July (winter, mean = 15 °C) and from 17 to 30 °C (mean = 23 °C) in January (summer), and the average monthly rainfall is 12.7 mm in July and 155.5 mm in January (data from CustomerWeather 2005–2015).

Observers were researchers, students, and volunteer field assistants trained to recognise all monkeys individually, and were tested for inter-observer reliability before they collected data. Opportunistic observations were made ad libitum during the course of other data collection at the project. Instances of dead infant carrying were recorded in daily logbooks and, in some cases, accompanied by videos, but mothers carrying deceased infants were not subject to focal follows.

The level of description of the cases varied, but for some observations, caretaking behaviours of the mother are detailed. Births were always recorded upon first sighting of a new baby; however, not every group was followed every day, and therefore the exact duration of carrying was not known for some cases. The pattern of group observation changed throughout the study period, but each group was generally observed at least 1 day per week throughout the year. We therefore report the minimum confirmed carrying time, as well as the maximum possible time. The latter measure was calculated as the time from when the female was last seen before carrying the dead infant to when she was seen carrying it, plus the time from when she was last seen carrying it to the next time she was seen without it. If the female was seen carrying the body on only 1 day, we report the confirmed duration as ≤ 1 day unless more detailed timing was available.

The study conforms with the Association for the Study of Animal Behaviour/Animal Behaviour Society guidelines for the care and use of animals. We used non-invasive observational methods of data collection on animals in their natural habitats, and all individuals were habituated to human observers.

## Results

Over a 10-year period, 14 instances of dead infant carrying were observed in females in six different groups in our study population (Table 1). The time spent carrying a dead infant (based on the most conservative estimates) ranged from approximately 50 min to 14 days. In seven cases, the females were confirmed to have carried the infant for less than 1 day. Five further females were directly observed carrying their deceased infants for ≤ 1 day, although due to gaps in observation, it is possible that they carried the infants for longer (between 2 and 4 days; see Table 1). Another female (Rosemary; Table 1) was observed carrying her deceased infant for a minimum of 2 days, and may have carried it for up to 3 days. One female was observed carrying her deceased infant for 14 days, although it is likely that she carried it longer, judging by the condition of the body when it was first observed (see case 1: Beminde). Eight cases involved infants 2 days old or less. Since the beginning of habituation in each group, a total of 390 births have been recorded. Of these, 97 infants have either died or disappeared (presumed dead) during their first year of life. Dead infant carrying was thus observed in 14.4% (14) of recorded infant deaths. For the remaining 85.6% of infant deaths, we were unable to confirm whether the infant was abandoned immediately, without this being witnessed by observers, or if carrying was otherwise prevented, for example due to predation.

**Table 1:** Details of each case of dead infant carrying observed between 2010 and 2019 at the Inkawu Vervet Project, Mawana Game Reserve, South Africa

Female	Group	Date first seen carrying corpse	Age of infant at death (days)	Confirmed minimum duration (days, unless stated otherwise)	Maximum possible duration (days, unless stated otherwise)	Mother’s first baby	Additional behaviours recorded
Hamba	AK	10 November 2013	38	≤ 1	2	No	
Beminde	IN	3 October 2014	< 9	14	27	No	Smelling, grooming, swatting flies from corpse; corpse left on ground while mother forages and frequently looks back at corpse’s location
Rosemary	LT	2 November 2015	< 2	2	3	Yes	Grooming corpse
Zeia	KB	3 November 2015	19	≤ 1	4	No	Grooming corpse; corpse left on ground while mother forages and frequently looks back at corpse’s location
Geleza^a^	AK	7 March 2016	103	≤ 1	1	NA	
Oahu	CR	10 October 2017	< 1	≤ 1	3	No	
Geneva	NH	5 November 2018	< 1	≤ 1	1	No	
Nyanga	AK	24 December 2018	1	≤ 1	3	Yes	
Nurks	BD	2 April 2019	140	≤ 1	1	No	Contact calling (after dropping the corpse)
Xalapa	NH	21 January 2019	< 1	≤ 1	1	No	
Gaya	NH	21 January 2019	13	≤ 1	2	No	
Numbies	BD	3 October 2019	< 1	≤ 1	≤ 1	No	Corpse left on ground while mother forages
Nessie	KB	24 October 2019	< 1	50 min	50 min	No	Licking, grooming, partial eating of the corpse (tail)
Puolka	BD	25 October 2019	2	< 1	< 1	Yes	

The data show the minimum duration of carrying that could be confirmed by observation and, due to gaps in observation, the maximum possible duration of carrying

*NA* Not ascertained

^a^This individual was the older sibling, not the mother, of the dead infant she carried; the infant was carried on the day following their mother’s death

The mother’s mode of carrying the dead infant whilst travelling or foraging was to hold it in one hand and locomote tripedally (Supplementary video 1); no other modes of carrying were seen. Unfortunately, most cases are simply descriptions of the mother carrying the dead baby, with few details on behaviour. However, in some cases additional caretaking behaviours were reported. These behaviours included grooming, licking and smelling the remains (*n* = 4), and protective behaviours such as swatting flies away and frequently looking towards the body when it was not being carried (*n* = 2). One female, Nurks, was observed contact calling after she dropped her infant. Another mother, Nessie, was seen to eat the end part of the tail of the body before continuing care-taking behaviours. Three cases are especially interesting: one showing a prolonged period of dead infant carrying (case 1: Beminde), one showing dead infant carrying by an individual other than the mother (case 2: Geleza), and one showing partial cannibalism of the dead infant by the mother (case 3, Nessie). All three of these cases are described below in more detail.

### Case 1: Beminde

On 3 October 2014, Beminde was seen carrying the corpse of her infant. The corpse was reported to smell bad, thus the infant was assumed not to have died that day. Beminde’s group (IN) was last observed 9 days prior to this observation, and no baby was reported with her then. Therefore, the baby may have been dead for a maximum of 9 days when first observed.

**Fig. 1:**
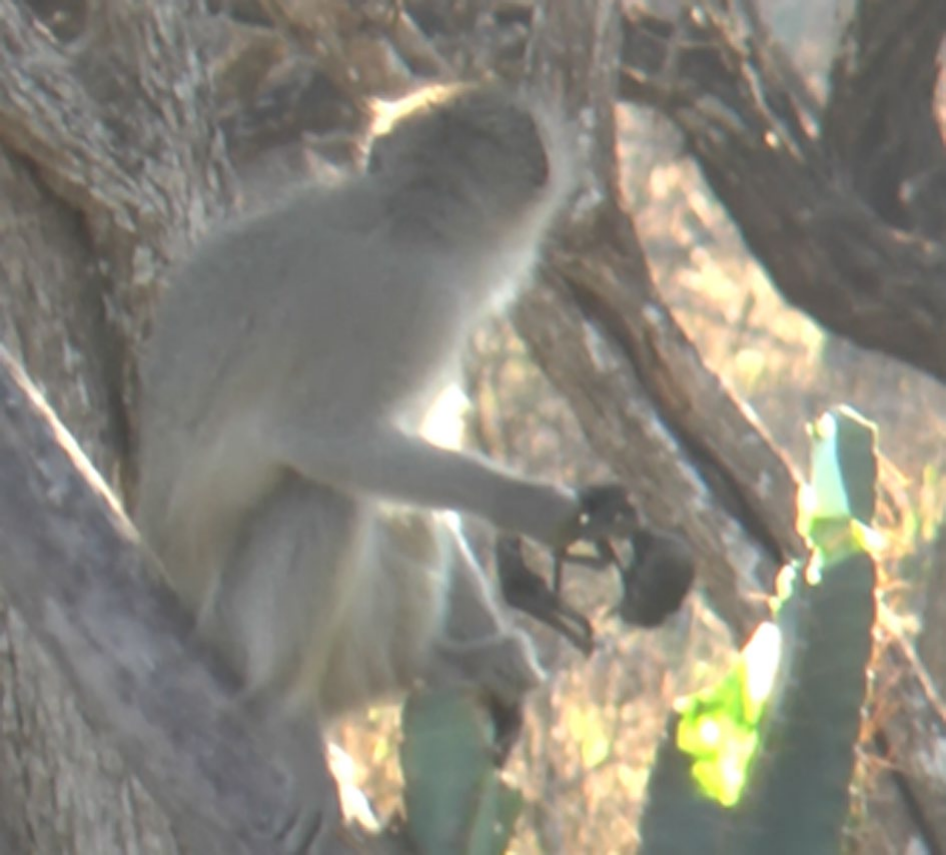
Beminde carrying the mummified remains of her infant. The photograph is a still from a video recorded by J. B. on 6 November 2014, three days after Beminde was first seen carrying her dead infant

Beminde was observed smelling and grooming the deceased infant, and carrying it in one hand. She was carrying the corpse on each of the three occasions that the group was observed over the next 14 days. By 6 October, the corpse appeared somewhat mummified, with its skeleton intact and its skin dried out (Fig. 1). Beminde held it in one hand and travelled tripedally (Supplementary video 2). She also held the corpse in one hand whilst foraging, and on at least one occasion she dropped it to forage with both hands. When this happened she glanced frequently at the corpse on the ground, before picking it up again after a few minutes. She picked up the corpse and smelled it before taking it with her as she moved, a behaviour that mothers sometimes direct at their live infants (personal observation). Several bouts of grooming and swatting flies away from the corpse were observed. Beminde was last seen carrying the corpse on 17 October, and when next seen, on 21 October, she was no longer carrying it.

### Case 2: Geleza

Geleza, a subadult female (3.5 years old and nulliparous), was seen carrying the body of her deceased infant maternal sibling. On 7 March 2016, Geleza’s mother, Gaga, died during or following a between-group encounter, the noise of which seemed to have attracted feral/village dogs. Soon after the encounter, Gaga was seen dead with extensive wounds, likely from dog bites, but possibly also due to vervet monkey aggression during the between-group encounter. At the time of this event Gaga had an approximately 3-month-old infant, Geleza’s younger sibling. The following day, Geleza was carrying the infant’s dead body when observers arrived at the group at approximately 5:40 a.m. The group left the reserve (and thus were not followed) at 9 a.m. When they were next seen on the reserve, at 2:30 p.m. on the same day, Geleza was no longer carrying her dead sibling. It is unknown if the infant died before or after Geleza started carrying it, or if another monkey carried it after Gaga died; however, no other group member was observed carrying it.

### Case 3: Nessie

In the early morning of 24 October 2019, adult female Nessie was observed giving birth while in a tree. The infant was either stillborn or died shortly after birth. Nessie’s 1-year-old son, Nugget, was near to her in the tree during the birth. Nessie was observed to drop the corpse of the infant to the ground, after which she climbed down, approached the corpse, and started to eat the placenta and part of the infant’s tail. She chewed on the tip of the tail, biting off no more than a quarter of its length. Observers did not see her spit anything out and therefore concluded that she consumed the end of the tail. Nugget moved ca. 20 m away from Nessie shortly after she began to eat the placenta, and began alarm calling. Nessie then carried the corpse into a tree where Nugget re-joined her. She then began to lick the corpse for around 20 min, before grooming its tail. Finally, approximately 50 min after the infant was born, Nessie dropped it for the second time and moved away. The corpse landed on a branch in the tree and was observed at the same spot the following day. No other monkeys were seen interacting with the corpse.

## Discussion

We report 14 cases of dead infant carrying by female vervet monkeys at the Mawana Game Reserve, South Africa, with particular reference to one case of carrying by the mother that lasted at least 14 days, one case of a sub-adult female carrying her dead sibling, and one observation of partial maternal cannibalism.

Overall, the patterns of dead infant carrying seen in this population of vervet monkeys appear similar to those seen in other monkeys, such as Japanese macaques at Takasakiyama (Sugiyama et al. 2009) and geladas at Guassa, Ethiopia (Fashing et al. 2011). In all three of these populations, most mothers carried their dead infants for between 1 and 3 days, with only a small number carrying them for more than 10 days. Furthermore, gelada non-mothers were also witnessed carrying dead infants for several hours (Fashing et al. 2011). In the macaque population, 15% of infants that died were carried (Sugiyama et al. 2009), which is very similar to the 14.4% of dead infants that were carried in our population of vervet monkeys.

The observed behaviour of Beminde, who carried her infant for at least 14 days, and likely several days more, provides another example of the relatively infrequently reported phenomenon of extended carrying of dead infants in primates. Of the 110 cases reviewed in Gonçalves and Carvalho (2019, Appendix S1), only nine lasted for 14 days or more, although Georgiev et al. (2019) also described a recent case in Zanzibar red colobus. It seems possible that cases of extended carrying are not as rare as initially thought, thus further study may see more reported. It has been suggested that extended carrying occurs especially in places with more extreme climates (e.g. the habitats of mountain gorillas and geladas), or during dry seasons (Biro et al. 2010), the conditions of which may slow the decomposition of a body (Fashing et al. 2011), although a recent analysis found no evidence that chimpanzees carried infant corpses for longer in the dry season (Lonsdorf et al. 2020). Beminde’s infant was born relatively early in the birth season (October), when climatic conditions were fairly dry compared to later in the season (November–December), which may have helped mummify the body.

Caring for a dead infant, and especially carrying it in one hand whilst walking or climbing using only three limbs (Supplementary video 1), likely incurs a significant energetic cost to the carrier [although Takeshita et al. (2020) found no evidence of energetic costs of dead infant carrying in a Japanese macaque mother]. We still do not fully understand the reasons for this behaviour, although several hypotheses have been proposed (Watson and Matsuzawa 2018). Some researchers have suggested the influence of maternal hormones (Biro et al. 2010; Anderson 2016). The post-birth release of hormones that promote mother-infant bonding may cause a mother to continue to provide maternal care even when her infant dies (Kaplan 1973). However, we also observed carrying of a dead infant by an older sub-adult sister (case 2: Geleza). Birth-related hormones cannot explain this case, although the mechanisms through which siblings bond may be a factor. Dead infant carrying by non-mothers has also been reported for geladas (Fashing et al. 2011), gorillas (Warren and Williamson 2004), chimpanzees (Lonsdorf et al. 2020) and baboons (Carter et al. 2020), although Carter et al. (2020) note that carrying by non-mothers is usually of short duration, as in our sibling vervet monkey. The learning to mother hypothesis has been proposed to explain why some non-mothers carry dead infants: behaving maternally towards even a dead infant can contribute toward gaining skills required to be a competent mother (Warren and Williamson 2004). Another hypothesis centres on stress reduction; in baboons, the loss of close kin has been shown to result in high levels of stress (Engh et al. 2006), and it has been suggested that the carrying of dead infants may be a coping mechanism to manage this stress (Takeshita et al. 2020). Both the learning to mother and stress reduction hypotheses should apply to both mothers and other kin, and may explain our observations. In Geleza’s case, her mother and sister were both killed in a violent interaction the day before, and it is possible that Geleza carried the latter’s body as a coping mechanism (cf. Takeshita et al. 2020).

Some researchers have also suggested that mothers may be unaware or unsure that their infants are dead, and so continue to provide maternal care (Watson and Matsuzawa 2018). However, behaviours directed at deceased infants that are not normally directed at live infants, such as carrying them slung across their backs (observed in chimpanzees and baboons), appear to contradict this (Biro et al. 2010; Gonçalves and Carvalho 2019; Carter et al. 2020; Lonsdorf et al. 2020). Our observations neither support nor refute this hypothesis, although the severity of decay of Beminde’s deceased infant suggests that unawareness of death may not fully explain continued care-taking by vervet mothers. Furthermore, our observation of partial cannibalism of a dead infant by the mother (case 3: Nessie) is striking because this behaviour is often accompanied by contradictory caregiving behaviours (see Watson and Matsuzawa 2018). Nessie was observed to eat part of the dead infant’s tail and then proceed to lick and groom the corpse. This cannibalistic act might suggest awareness that her infant was dead, but then why would Nessie go on to perform care-taking behaviours? Seemingly rare instances of maternal cannibalism have been reported in other primate species including Tonkean macaques (de Marco et al. 2018), chimpanzees (Fedurek et al. 2020), and, at two different sites, bonobos (Tokuyama et al. 2017). To our knowledge, maternal cannibalism has never previously been reported in vervet monkeys, and this is the only case recorded in 10 years of observations at our field site.

Finally, it has been hypothesized that infant age at death might influence the mother’s response. Again, hormones may play a role in this, and stronger attachment may lead to stronger responses after infant death (Kaplan 1973; Anderson 2016). One prediction is that the death of older infants should cause stronger post-death responses in mothers, although a recent analysis of chimpanzee infant corpse carrying found no relationship between infant age at death and duration of carrying (Lonsdorf et al. 2020). In our sample, most cases involved newborns; the few older infants that died were not carried for longer than newborns, although our sample is too small to draw firm conclusions from this. We include details of infant age at death here so that the hypothesis can be more fully addressed in the future along with other hypotheses relating to individual differences in corpse carrying.

## Electronic supplementary material

Below is the link to the electronic supplementary material.

Supplementary video 1: Female, Nyanga, from AK, travelling tripedally while carrying her deceased infant in her right arm on 24 December 2018. Credit: Fady-Abbes Al-Shemmery (MP4 13635 kb)

Supplementary video 2: Female, Beminde, from IN, holding her deceased infant, which had been dead for at least 3 days, but likely longer, on 6 October 2014. Credit: Jennifer Botting (TS 102505 kb)
